# Thermal Boundary Resistance Reduction by Interfacial Nanopatterning for GaN-on-Diamond Electronics Applications

**DOI:** 10.1021/acsaelm.5c00119

**Published:** 2025-03-27

**Authors:** Xiaoyang Ji, Sai Charan Vanjari, Daniel Francis, Jerome A. Cuenca, Arpit Nandi, David Cherns, Oliver A. Williams, Felix Ejeckam, James W. Pomeroy, Martin Kuball

**Affiliations:** † Centre for Device Thermography and Reliability (CDTR), University of Bristol, Bristol BS8 1TL, U.K.; ‡ Akash Systems, San Francisco, California 94108, United States; § Cardiff School of Physics and Astronomy, Cardiff University, Cardiff CF24 3AA, U.K.

**Keywords:** GaN-on-diamond, thermal boundary resistance, thermoreflectance, nanopatterning, thermal simulation

## Abstract

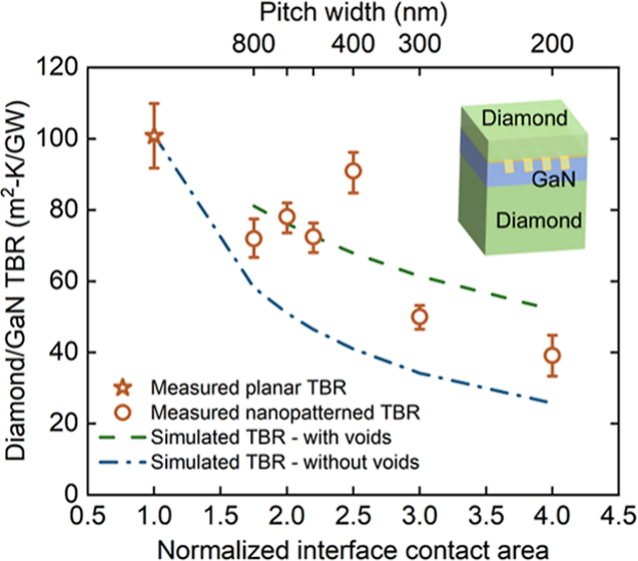

GaN high electron mobility transistors (HEMTs) on SiC substrates are the highest performing commercially available transistors for high-power, high-frequency applications. However, Joule self-heating limits the maximum areal power density, i.e., operating power is derated to ensure the lifetime of GaN-based devices. Diamond is attractive as a heat sink due to its record-high thermal conductivity combined with its high electrical resistivity. GaN-on-diamond devices have been demonstrated, bringing the diamond as close as possible to the active device area. The GaN/diamond interface, close to the channel heat source, needs to efficiently conduct high heat fluxes, but it can present a significant thermal boundary resistance (TBR). In this work, we implement nanoscale trenches between GaN and diamond to explore new strategies for reducing the effective GaN/diamond TBR (TBR_eff_). A 3× reduction in GaN/diamond TBR_eff_ was achieved using this approach, which is consistent with the increased contact area; thermal properties were measured using nanosecond transient thermoreflectance (ns-TTR). In addition, the SiN_*x*_ dielectric interlayer between the GaN and diamond increased its thermal conductivity by 2× through annealing, further reducing the TBR. This work demonstrates that the thermal resistance of heterogeneous interfaces can be optimized by nanostructured patterning and high-temperature annealing, which paves the way for enhanced thermal management in future device applications.

## Introduction

Ever increasing areal power density and device miniaturization are required for the continued development of power and radio frequency semiconductor electronics, electric vehicles, wireless communications, and satellite communications.^1−6^ Gallium nitride (GaN) high electron mobility transistors (HEMTs) have gained a significant market share in RF electronics due to their high critical electric field, high electron mobility, and high electron saturation velocity.^1−6^ Emerging device technologies such as fin field-effect transistors (FinFETs) are also receiving increasing attention.^7−10^ Self-heating in GaN-based devices, however, cannot be ignored; it occurs in the channel on a submicron length scale due to Joule heat generation.^11,12^ If not carefully managed, the resulting channel temperature rise can reach >200 °C induced by high heat fluxes around the hot spot location, which ultimately degrades performance and accelerates time to failure.^12,13^ Operating voltages, current, or areal power density must be derated to mitigate this, preventing GaN from reaching its ultimate performance potential. Therefore, improving near-channel heat extraction in GaN-based devices is critical to maintaining excellent performance and ensuring a safe lifespan. Diamond is the ultimate heat spreading material, having the highest thermal conductivity of any bulk material. Diamond can be integrated as the substrate (GaN-on-diamond) or top side heat spreading coating (diamond-on-GaN) within ∼1 μm of the channel hot spot to achieve an impressive reduction in thermal resistance.^14,15^ Nevertheless, diamond integration can be improved further by reducing the effective thermal boundary resistance (TBR_eff_) associated with the GaN/diamond interface, which is known to be a thermal bottleneck due to the tremendously high heat fluxes close to the channel heat hot spot.^12,16^

The integration of GaN and diamond can be achieved in four ways: (1) growth of GaN on a diamond substrate; (2) bonding between diamond and GaN; (3) growth of diamond on the back side of GaN; (4) growth of diamond on the front side of GaN.^17^ As for the first two methods, the direct growth of GaN on the diamond substrate typically requires a thick AlGaN transition layer resulting in a high thermal resistance; on the other hand, bonding techniques still faces challenges in achieving good uniformity and robust bonding for large GaN-on-diamond wafers.^17^ Therefore, the direct growth of diamond on GaN is considered to be the most scalable method: Removing the Si substrate from a GaN-on-Si wafer, and directly depositing diamond with a thin protective, adhesion promoting SiN_*x*_ or AlN dielectric layer.^17,18^

The GaN/diamond effective TBR (TBR_eff_) is a lumped resistance including the TBRs of each interface, the thermal resistance of the dielectric interlayer, and the diamond nucleation layer. TBR_eff_ greatly depends on the quality of the interlayer and the initial diamond nucleation. The direct deposition of diamond onto GaN without any interlayer has a high effective TBR because of the lack of carbide bond formation.^19^ van der Waals bonded interfaces can have a TBR as high as 220 m^2^ K/GW.^20−23^ Therefore, a dielectric layer (SiN_*x*_ or AlN) is commonly used to form a strong carbide bond, enabling efficient phonon transmission across the interface.^24−26^ Phonon transmission may also be enhanced by bridging the gap in phonon density of states between diamond and GaN, e.g., using SiC, AlN, or SiN_*x*_ interlayers.^14,16,27^ Thus, adding a dielectric layer can decrease TBR_eff_.^16,27^ Previous reported measured GaN/diamond TBR_eff_ values vary widely depending on the interlayer material and the diamond nucleation method; with an amorphous AlN or SiN_*x*_ dielectric layer with ≤50 nm thickness, TBR_eff_ can range from 5 to 100 m^2^ K/GW,^21−23,28−34^ dependent on the thickness of the dielectric layer and its material quality.^22,31,35^ The GaN/diamond TBR_eff_ is proportional to the thickness of the interlayer, and extrapolating to 0 nm interlayer thickness (no interlayer) reaches as low as 3 m^2^ K/GW, similar to the diffusion mismatch model (DMM) TBR prediction, which is the minimum limit.^17,36^ However, in reality, this low value cannot be reached because, as discussed previously, diamond deposition without an interlayer will have weak adhesion without a carbide forming interlayer. Furthermore, the interlayer protects the GaN surface during the first stage of diamond growth, preventing pin holes being etch into the GaN; a minimum interlayer thickness is therefore required.^37,38^

An alternative approach for reducing GaN/diamond TBR_eff_ is investigated here: Increasing the contact area between the GaN and diamond. Densifying the interlayer by high-temperature annealing to improve the TBR_eff_ is also studied. To achieve this, a corrugated pattern was fabricated at the GaN/diamond interface, a dielectric interlayer deposited followed by annealing before diamond deposition. The GaN/diamond TBR_eff_ is assessed by using nanosecond transient thermoreflectance (ns-TTR). The GaN/diamond interface nanostructures are observed by transmission electron microscopy (TEM), and the interface composition is determined through X-ray energy-dispersive spectroscopy (EDS) mapping. The TBR_eff_ improvement versus the contact area is compared with thermal simulation results. Improving TBR_eff_ will contribute to the advancement of the next generation of ultrahigh power density RF components by overcoming a key interface thermal bottleneck.

## Experimental Details

### Sample Preparation

First, SiN_*x*_ films with thicknesses of 25 nm or less were deposited onto unetched GaN/diamond wafers at 300 °C using PECVD. These served as reference TBR_eff_ benchmark and assisted us to study how the SiN_*x*_ TBR_eff_ is affected by SiN_*x*_ densification. Then samples were rapidly thermally annealed at 600, 800, and 1000 °C for 10 min under a N_2_ atmosphere.

**Figure 1:**
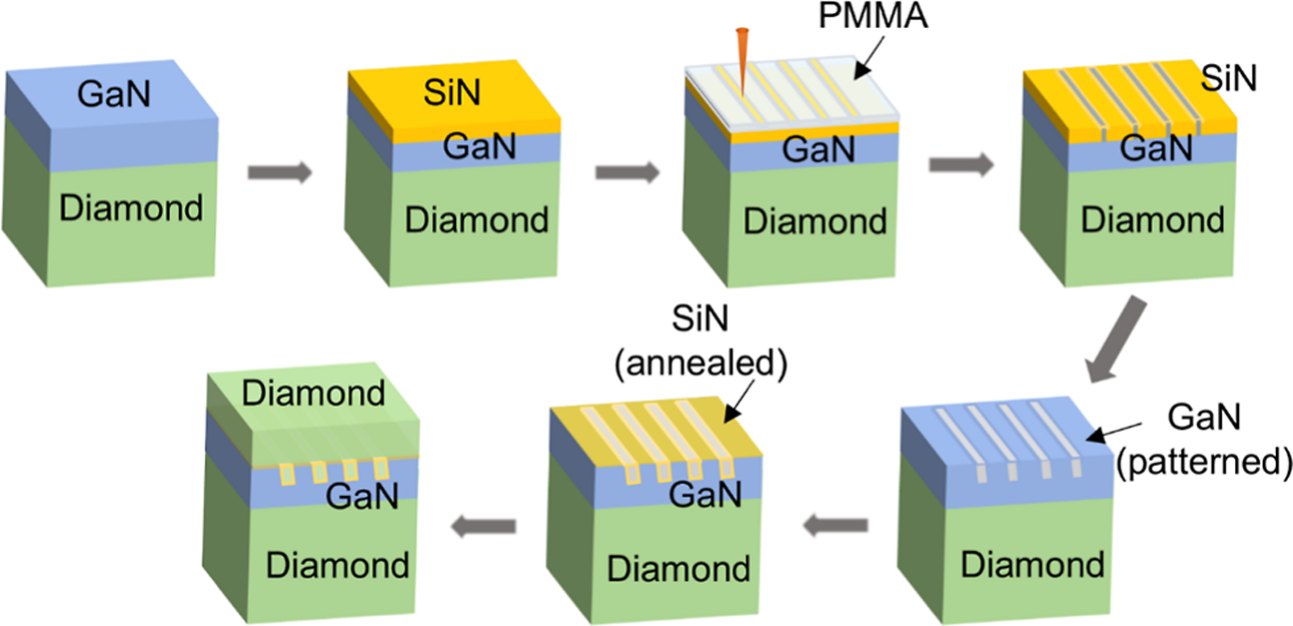
Process flow for patterning at the diamond/GaN interface (not to scale).

To fabricate the patterned surface illustrated in Figure 1, an ∼50 nm-thick SiN_*x*_ thin film was deposited using plasma-enhanced chemical vapor deposition (PECVD) on a GaN/diamond wafer, with a 750 nm thick GaN layer, to serve as a hard mask. A poly(methyl methacrylate) (PMMA) layer was coated on top of the SiN_*x*_; e-beam lithography was used to pattern trenches into the PMMA layer; then the trench patterns were transferred into the SiN_*x*_ using an SF_6_ plasma by reactive ion etching (RIE). The underlying GaN was etched through the trenches in the SiN_*x*_ hard mask using RIE with Cl_2_/Ar plasma. The PMMA and SiN_*x*_ layers were then removed. Etched GaN trench widths were fixed as 100 nm and the pitch width (the period of the pitch, i.e., distance from one trench center to another trench center) was 800, 600, 500, 400, 300, and 200 nm. Numerous cells were created on the wafer, with each unit cell featuring identical pitch patterns and etched trenches. The relative contact area between GaN and diamond for the pitch widths of 800, 600, 500, 400, 300, and 200 nm is 1.75, 2, 2.2, 2.5, 3 and 4, respectively (assuming 300 nm trench depth), with 1 denoted as contact area for a planar unpatterned interface. A 10 nm PECVD SiN_*x*_ layer was subsequently conformally deposited and rapid thermally annealed at 1000 °C, followed by a 1 μm diamond thin film deposition by microwave plasma-assisted chemical vapor deposition (MPCVD).

Diamond was nucleated by immersing the sample in a nanodiamond colloid solution containing particles with positive zeta-potential under ultrasonic agitation, followed by drying with N_2_.^39^ The sample was mounted on a custom MPCVD sample holder to regulate the sample temperature to ∼700 °C using a Carat Systems CTS6U at 5 kW and 160 mbar in a 3% CH_4_/H_2_ gas flow at 300 sccm for 1 h.^40^ The substrate temperature was monitored using dual wavelength pyrometry and calibrated using a silicon substrate of the same thickness. It is worth noting that the diamond was deposited on top of GaN to facilitate thermal measurements with high TBR sensitivity. However, in practical applications, this nanopatterned interface would only be applied between the backside of the GaN and the diamond substrate, to avoid damaging the two-dimensional electron gas (2DEG) close to the surface that forms the transistor channel.

### Thermal Characterization

The TBR_eff_ values of annealed SiN_*x*_ and the nanopatterned diamond/GaN interface were measured using ns-TTR. A 10 nm Cr adhesion layer and a 160 nm Au transducer layer were coated onto the SiN_*x*_/GaN/diamond and the diamond/GaN/diamond structure via thermal evaporation prior to the measurement. The ns-TTR setup is similar to those in previous reports, and its apparatus is shown in Figure S1.^41^ The 532 nm pulsed pump laser heats the metal transducer layer with a pulse repetition frequency of 8 kHz and a 3 ns pulse full-width-of-half-maximum (fwhm). A polarizing beam splitter was used to split a 488 nm continuous wave (CW) probe laser into a reference beam and another beam reflected from the sample measuring the thermoreflectance signal. Both beams were directed onto a balanced photodetector having comparable path length, canceling out the noise signal with the thermoreflectance signal remaining. The output signal was recorded using an oscilloscope with a 1 μs time window around the pulse; 65,534 waveforms were averaged per acquisition to improve the signal-to-noise ratio (SNR). Each measurement was repeated three times and averaged to further improve the SNR.

Since the waveforms were oversampled in the longer time scale portion of the measured transient, a digital low pass filter was applied with logarithmic time spacing to further improve the SNR. The resulting transient curve was fitted by using a numerical solution of the axisymmetric multilayer heat diffusion model. The thermal conductivity of GaN was determined from the reference planar GaN/diamond structures and then fixed for the analysis of subsequent samples. The thermal properties of the materials in the SiN_*x*_/GaN/diamond and the diamond/GaN/diamond structure are listed in Tables S1 and S2 in the Supporting Information, respectively. Sensitivities of interested parameters were calculated in the typical way
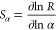
1where *R* is the normalized signal and α is the thermal property of interest.^41^

## Results and Discussion

**Figure 2:**
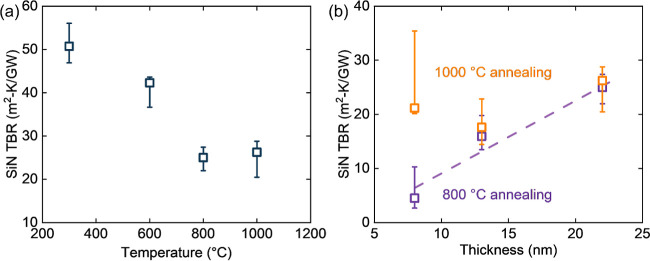
(a) TBR_eff_ change of SiN_*x*_ thin films deposited on GaN/diamond substrates before (deposited at 300 °C) and after rapid annealing (600 °C, 800 °C, and 1000 °C). (b) TBR_eff_ of SiN_*x*_ thin films with different thicknesses after 800 and 1000 °C annealing. The upper and lower bounds of data points are based on the data fitting within confidence.

Figure 2a shows how annealing temperature and SiN_*x*_ thickness affect the TBR_eff_ of the reference (unpatterned) SiN_*x*_ thin films deposited on GaN/diamond substrates; a detailed description of the analysis is given in the Supporting Information. SiN_*x*_ TBR_eff_ demonstrates a reduction by a factor of 2 after rapid annealing at 800 °C, with a 1000 °C annealing yielding no further improvement. Specifically, the TBR_eff_s of the 22 nm SiN_*x*_ thin films were determined to be 51, 42, 25, and 26 m^2^ K/GW, as deposited at 300 °C and annealed at temperatures of 600, 800, and 1000 °C, respectively. This is consistent with previous observations that rapid thermal annealing densifies SiN_*x*_, resulting in the formation of stoichiometric Si_3_N_4_.^42,43^ The relationship between TBR_eff_ and SiN_*x*_ thicknesses is shown in Figure 2b for samples annealed at 800 or 1000 °C. For 800 °C, there is a linear relationship between TBR_eff_ and SiN_*x*_ thickness, as expected from previous studies: 5, 16, and 25 m^2^-K/GW at thicknesses of 8, 13, and 22 nm, respectively. The thermal conductivity of SiN_*x*_ samples annealed at 800 °C is ∼1.2 W/m K (averaged from the three samples with different thicknesses), which is consistent with previous measurements of amorphous silicon nitride thin films.^31^ However, the TBR_eff_ of the SiN_*x*_ annealed at 1000 °C is more or less constant regardless of thickness, with an average value of 22 m^2^ K/GW.^17,36^ This suggests that the SiN_*x*_ thermal conductivity decreased with reduced film thickness after annealing at 1000 °C, and SiN_*x*_ films thinner than 22 nm degrade at this annealing temperature. Thinner amorphous SiN_*x*_ films annealed at 1000 °C may develop more material defects, likely due to diffusion, leading to increased phonon scattering and reduced thermal conductivity. The differing TBR_eff_ dependence on SiN_*x*_ thickness at 800 and 1000 °C is attributed to variations in SiN_*x*_ film material quality.

**Figure 3:**
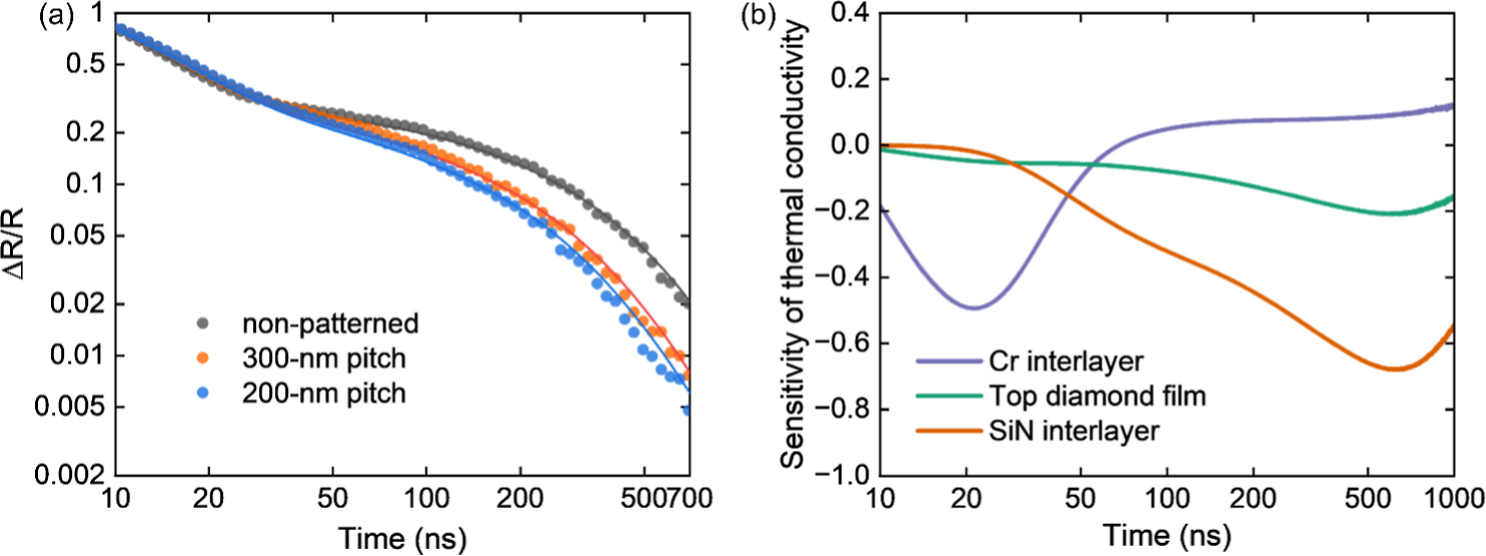
(a) Normalized transient curves from ns-TTR measurements for the diamond/GaN/diamond samples with interfaces without a pattern, with a 300 nm pitch pattern, and a 200 nm pitch pattern. The dots indicate experimental data, while the lines are the fitting results. (b) Sensitivity analysis of ns-TTR measurements on diamond/GaN TBR_eff_. In the sensitivity analysis, Λ_Cr_ = 0.42 W/m K, thickness *t*_Cr_ = 10 nm; Λ_topdiamond_ = 200 W/m K, *t*_topdiamond_ = 850 nm; Λ_SiNx_ = 0.31 W/m K, thickness *t*_SiNx_ = 10 nm; the other parameters for sensitivity analysis are given in Table S2.

Building on the understanding of TBR_eff_ reduction through SiN_*x*_ densification by high-temperature annealing, we then studied the impact of the nanopatterned interface on TBR_eff_ using the diamond/GaN/diamond structures. In this work, the results with SiN_*x*_ interlayer between top diamond and GaN annealed under 1000 °C are reported, although the SiN_*x*_ interlayer with 8 nm thickness under 800 °C annealing has the lowest SiN_*x*_ TBR_eff_. However, the relatively high SiN_*x*_ TBR_eff_ increases the sensitivity of the diamond/GaN TBR_eff_ in thermal measurements, which is good for studying TBR_eff_ change by the impact of nanopatterning. Figure 3a shows the measured ns-TTR transients for diamond/GaN/diamond structures with 300 and 200 nm GaN-trench pitch, benchmarked against planar diamond/GaN/diamond structures. The temperature (proportional to reflectivity change) decays more quickly for the patterned samples, indicating the patterned interface does reduce the thermal resistance. Figure 3b shows the sensitivities of the TTR traces to the thermal conductivities of the Au/diamond interlayer (Cr), the top diamond film, and the diamond/GaN interlayer. The thermal conductivity of the Cr adhesion layer, i.e., the Au/diamond TBR, predominantly influences the fitting of the transient curve from 10 to 40 ns. Meanwhile, the diamond/GaN interface TBR_eff_ is the dominant fitting parameter from 200 to 1000 ns, with at most 4× larger sensitivity compared to that for the 1 μm diamond thermal conductivity; the fitting process is described in the Supporting Information. Therefore, the fast thermoreflectance decay in the 200–1000 ns time frame indicates that TBR_eff_ is reduced for the patterned interfaces.

**Figure 4:**
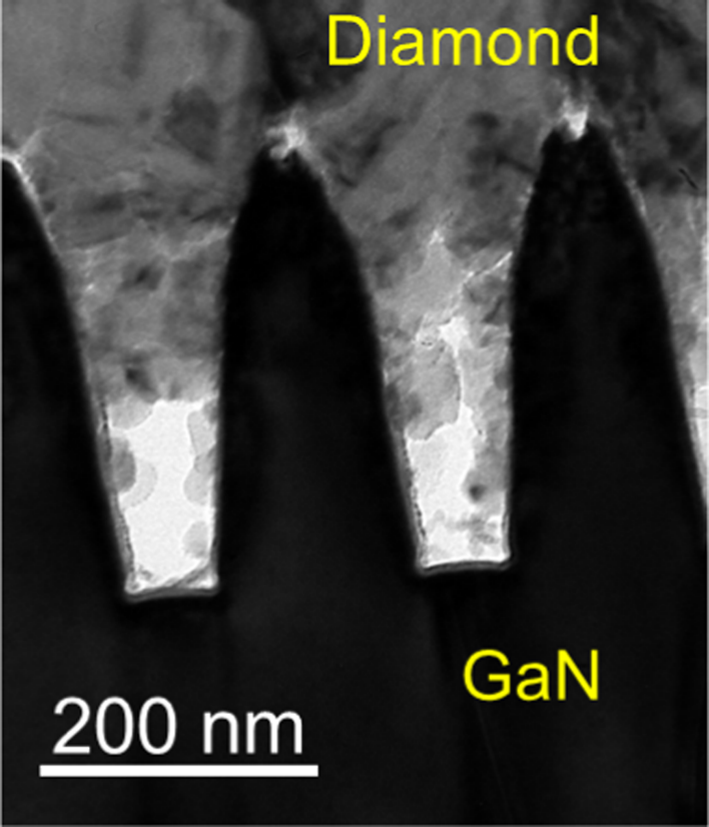
Cross-sectional transmission electron microscopy (TEM) micrograph of the diamond/GaN/diamond structure. The GaN trench bottoms are rectangular, but the side walls are concave, making trenches wider at the top than at the bottom. The diamond generally does not fully fill the GaN trenches, with voids near their bottom region. See Figure S3 for other examples.

TEM and EDS were used to investigate the microstructure and composition of the patterned interfaces. Cross-sectional lamella were produced by ThermoFisher Scios 2 DualBeam focused ion beam (FIB) milling from the sample with a 200 nm pitch. A 30 kV, 30 nA, and 30 kV, 15 nA chunk cutting sequence was used, followed by cleaning at 30 kV 3 nA and 30 kV 50 pA, thinning of the lamellae at 30 kV 0.5 nA and 30 kV 0.3 nA, and polishing at 5 kV. Cross-sectional micrographs were obtained in a Philips CM30 transmission electron microscope, with a voltage of 300 kV. EDS mapping was performed in a JEOL TEM with a voltage of 200 kV. More details are supplied in the Supporting Information. Typical diamond/GaN interfacial regions are illustrated in Figures 4 and S3, showing the 200 nm pitch, with a trench depth of 320 nm, in reasonable agreement with aimed 300 nm. The GaN trench bottoms are rectangular, but the side walls are slightly concave, making trenches somewhat wider at the top than at the bottom. Trenches filled only between ∼20 and 80% with diamond, i.e., voids are present in the diamond. During MPCVD, the diamond seeds toward the top of the trenches coalesce, limiting growth at the bottom of the trenches due to the high aspect ratio of the trenches. EDS mapping in Figure S4 in the Supporting Information showed that the SiN_*x*_ thin film is approximately 10–20 nm thick, uniformly across the trenches, i.e., there was conformal SiN_*x*_ deposition.

**Figure 5:**
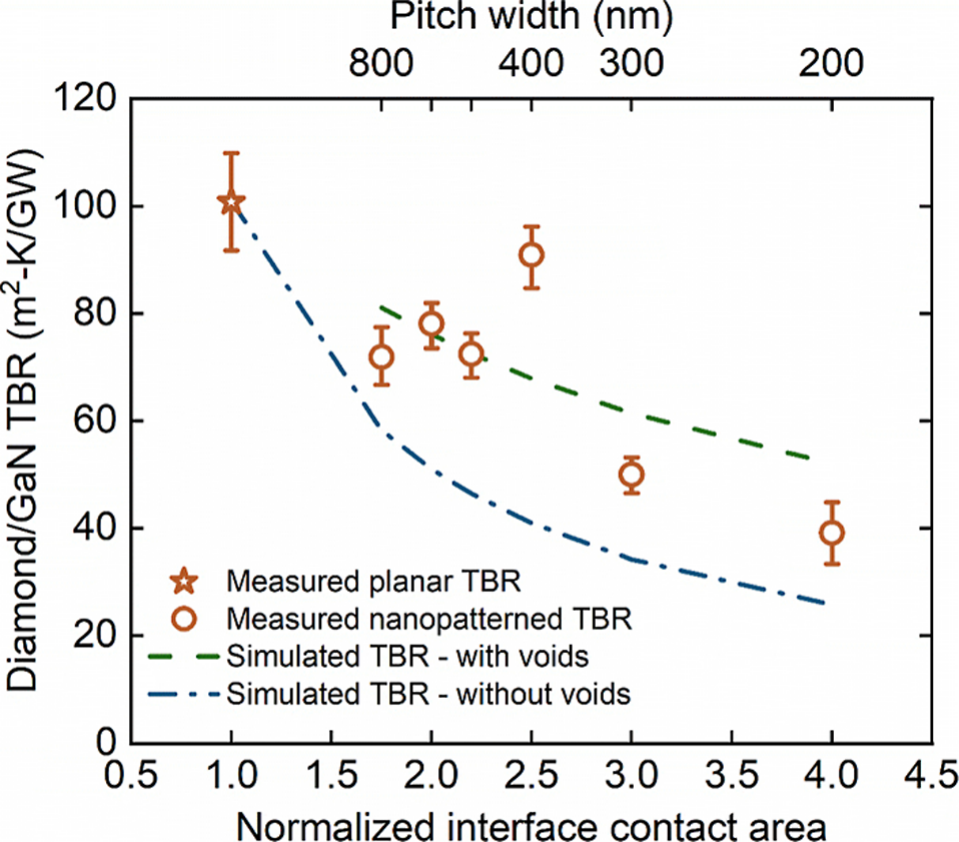
Measured diamond/GaN TBR_eff_ as a function of pitch width/ideal contact area; the upper and lower bounds of points are based on data fittings ns-TTR measurements. The TBR_eff_ of the planar and 200 nm pitch samples were measured at 2 and 3 different locations, respectively, and the upper and lower bounds included represent the standard deviation of the fitted TBR_eff_; other samples were measured at a single location and the bounds are from single-point data fitting within confidence. The measured TBR_eff_ is compared with finite-element thermal simulation predictions, considering trenches fully filled with diamond and partially filled (50%).

Figure 5 shows the measured diamond/GaN TBR_eff_ values for different pitch widths. In the cross section, the ideal interface contact area per period of the patterned structure is *A* = *l* + 2*d,* where *l* is the pitch width and *d* is the trench depth. However, the area is simply *l* for the unpatterned interface. The upper limit for the ratio of interface contact increase is therefore (*l* + 2*d*)/*l* from patterning. Smaller pitch widths correspond to a larger contact area between the top diamond and GaN through the interfacial densified SiN_*x*_; we normalized the ideal nominal contact area based on the unpatterned planar area (shown as 1 on the *x*-scale); then the relative interface contact areas are 1.75, 2.0, 2.2, 2.5, 3.0, and 4.0 if assuming ideal rectangular trenches and 300 nm trench depth, for pitches of 800, 600, 500, 400, 300, and 200 nm, respectively. Nanopatterning of the interface clearly provides great benefits in enhancing heat transport from the diamond into the GaN and vice versa. The TBR_eff_ values decrease from 101 m^2^ K/GW for the planar interface to 39 m^2^ K/GW for the interfaces with pitches of 200 nm, a decrease by a factor of 2.6× with a nearly 4-fold increase in contact area (200 nm pitch); this is despite the presence of voids in the diamond. There is a clear trend of decreasing TBR_eff_ with an increasing contact area.

Based on the TEM images in Figures 4 and S3, we can roughly estimate the actual interfacial contact area, considering the presence of voids. The diamond grains initially grow on the sidewalls of the GaN trenches and can partially or fully fill half of the trenches. As a result, diamond contacts the upper part of the trench sidewalls, to a depth of between 50% and 80%. In the worst-case scenario, where the diamond only fills the upper 50% of the trench sidewalls, the reduced cross-sectional contact area is *A*_reduced_ = *l* + 2*d*/2 – *w*, where *w* is the trench width. Taking the designed trench depth and width of 300 and 100 nm, respectively, the calculated ratio for the contact area increase, with respect to the unpatterned interface, for the pitches of 800, 600, 500, 400, 300, and 200 nm are 1.25, 1.33, 1.40, 1.50, 1.67, and 2.00, respectively. However, the best measured TBR_eff_ reduction is 2.6×, suggesting that on average the diamond fills >50% of the trench sidewalls in practice.

To estimate the impact of the voids, a steady-state finite-element thermal simulation using Ansys was employed. The simulation modeled a 2-dimensional multilayered unit cell under a periodic boundary condition with a diamond/GaN/diamond structure where GaN is etched in the ideal rectangular shape. A small heat flow was applied to the top diamond surface, and a fixed temperature boundary was applied to the bottom of the diamond substrate. The averaged temperature difference Δ*T* between the bottom of the diamond thin film (*T*_up_) and the top of the GaN trench (*T*_bottom_) was calculated to obtain the effective diamond/GaN TBR. Applying Fourier’s law, TBR_eff_ = *A* × Δ*T*/*P*, where *A =* pitch width × cell thickness represents the area of the interface perpendicular to the cross-plane direction in the studied cell of the thermal model and *P* is the total heat power across the interface in the thermal model.^24^ The material properties determined from the ns-TTR data analysis were used, as listed in Table S2.

**Figure 6:**
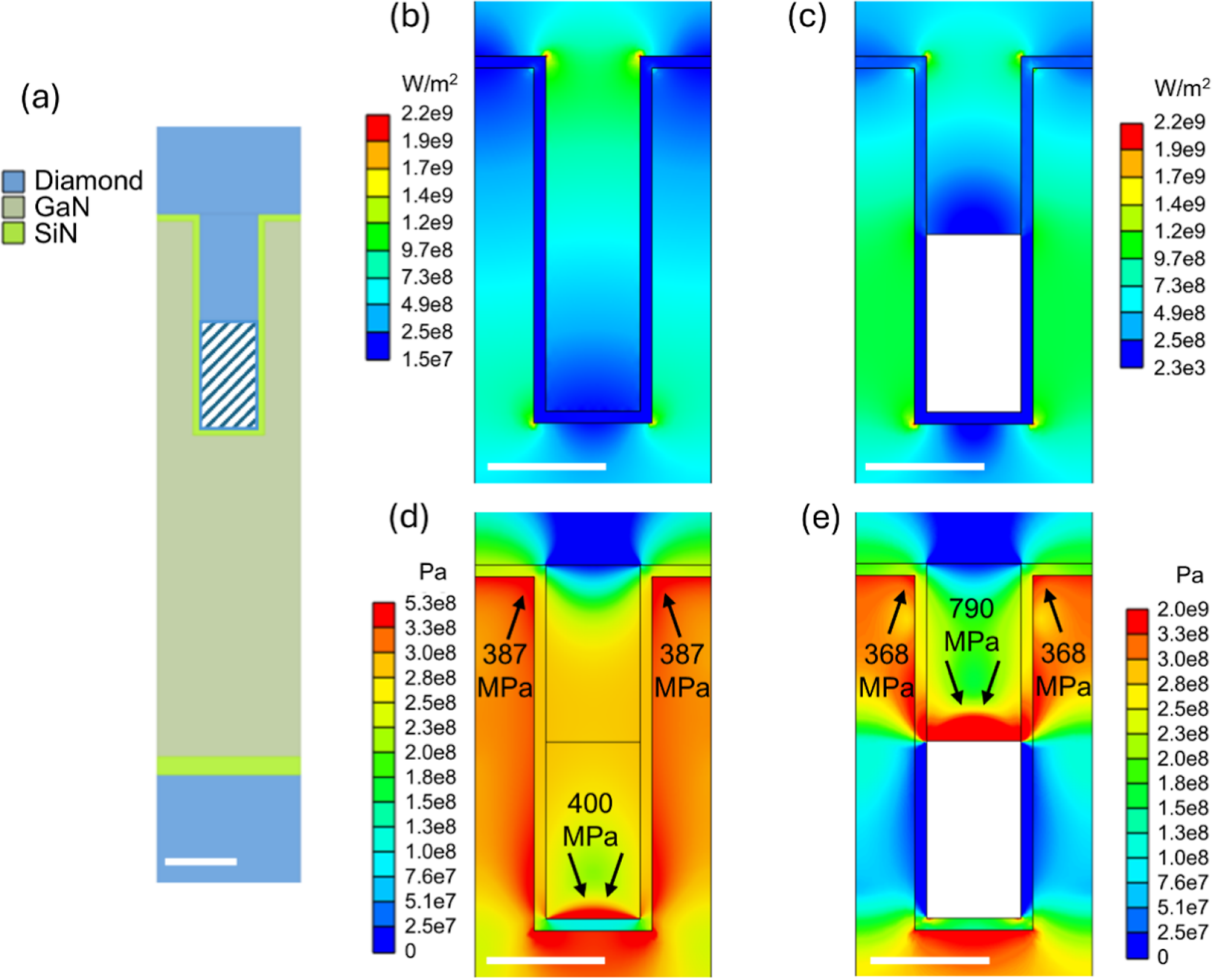
Finite element simulation of diamond filled GaN trench: (a) Diamond/GaN/diamond multilayered structure; pitch width considered is 200 nm; steady-state heat flux in (b) fully filled GaN trenches and (c) 50% filled trenches; stress due to mismatch of thermal expansion in (d) for fully filled trenches and (e) for 50% filled trenches. The scale bars are 100 nm. The maximum principal stresses are pointed out by arrows in (d) and (e). The highest value in the color bars in (d) and (e) may reflect simulation artifacts at vertices.

Assuming a 50% voiding and idealized trench shape, illustrated in Figure 6a, the TBR_eff_ predicted by the finite-element model is close to the measured trend versus contact area, reducing by 2.6× for the 200 nm sample with respect to the planar sample. Simulating the diamond/GaN TBR_eff_ for trenches completely filled with diamond overestimates the experimentally measured TBR_eff_ reduction; this represents the limiting case where contact area is inversely proportional to pitch, up to a 4× reduction. As illustrated in Figure 6, the heat flux from the steady-state thermal simulations for the fully filled and partially filled diamond/GaN pitches is compared in parts (b) and (c), respectively. The heat flux is concentrated at the top and bottom corners of the GaN trenches in the steady-state thermal models. In a previous study, the TBR_eff_ of nanopatterned diamond/Si interfaces was found to be inversely proportional to the contact area (the diamond/Si TBR_eff_ decreased by 65% following a 69% increase in contact area).^35^ Previous simulations of GaN/SiC, Al/GaN, and Al/Si nanopatterned interfaces also represented a TBR reduction proportional to the increased contact area.^44−47^ However, another reported diamond/Si sample with a 200 nm pitch width, 100 nm trench width, and 100 nm trench depth showed only a 26% TBR decrease, which was significantly lower than what is observed here. Thus, achieving a controlled TBR reduction through contact area adjustment can be challenging in experimental nanopatterning. The TBR_eff_ in this GaN-on-diamond work represents a greater reduction than the results in previous reports.^35^ It is noted that the previously experimental results, e.g., on diamond/Si structures, primarily focused on engineering corrugated interfaces, but the investigated structures are not particularly suited for practical GaN-based electronic applications. In comparison, this work furthermore determines the trend experimentally related to the GaN/diamond interfacial contact area, providing insights more directly relevant to the thermal management in GaN-based electronics applications with nanostructured interfaces. This aspect, particularly concerning future applications with ultrahigh thermal conductivity diamond substrates, has not been fully studied before.

Although significant TBR_eff_ reduction was achieved despite the voids in diamond, there may be a risk of fracture due to the thermo-mechanical stress at the corrugated diamond/GaN interface. We explored the stress in a 2-D Ansys finite-element model with periodic boundary conditions. The coefficients of thermal expansion, elastic modulus, and Poisson ratio of GaN, diamond, and SiN_*x*_ were taken from literature.^48,49^ Isothermal thermo-mechanical stress was simulated for both diamond filled trenches and trenches with voids in Figure 6d,e, respectively, assuming that the structure is relaxed at the 700 °C diamond deposition temperature and then cooled to room temperature; the maximum principal stress is as displayed, which is the typical failure criteria used for brittle materials which fracture.

As shown in Figure 6, the maximum principal stress for both fully diamond-filled and 50% diamond-filled GaN trenches is concentrated at the top corners of the GaN and the bottom of the diamond within the trenches. In fully filled trenches, the averaged stress at the GaN corners is 387 MPa over a 3 nm × 3 nm area (to avoid artificially high stress at corner vertex), while in partially filled trenches, this value decreases slightly to 368 MPa. This suggests that the GaN in partially diamond-filled trenches experience marginally lower thermal stress compared to the GaN in fully diamond-filled trenches, but both stress levels remain well below the tensile strength of nanostructured GaN (4–7 GPa).^50^ Moreover, the stress distribution at the bottom of the diamond (within the GaN trenches) also differs between the two cases, averaging around 400 MPa in fully filled trenches and approximately 790 MPa in partially filled trenches. Despite the higher induced thermal stress in the diamond of partially filled trenches, it is far below the compressive strength of CVD diamond, which exceeds 100 GPa.^49,50^ In conclusion, nanopatterning with trenches does not induce significant thermal stress-related damage during MPCVD, regardless of the trench filling level, but the TBR_eff_ reduction is still significant.

The TBR_eff_ of GaN/diamond can vary significantly, ranging roughly from 5 to 100 m^2^-K/GW, depending on the interlayer quality between diamond and GaN, although the intrinsic TBR of the GaN/SiN_*x*_ and SiN_*x*_/diamond interfaces can be as low as 3 m^2^ K/GW (formation of strong covalent bonds between these materials).^14,16,31^ In this work, the high TBR_eff_ of the diamond/GaN planar interface might be attributed to poor contact between the diamond and SiN_*x*_ during diamond growth, which could be due to insufficient diamond nucleation or imperfect seeding density. Furthermore, TBR_eff_ reduction by nanopatterning due to a contact area increase might be impeded by other factors other than voiding. First, simulations have shown that different trench shapes can slightly affect the TBR. The triangular trench shape leads to a higher TBR because of the less contact area compared to the rectangular shape.^46^ Furthermore, the high aspect ratio for narrow trenches, e.g., with 100 nm trench width, might limit the maximum diamond grain size achievable, i.e., reduce the thermal conductivity of the diamond, resulting in a higher effective TBR. Therefore, efficient TBR reduction in nanopatterning might be further improved by further optimizing trench shapes and more optimized aspect ratio of the trenches, in addition to an improved diamond filling of the trenches during growth.

## Conclusions

Self-heating in GaN-based electronics poses a significant obstacle to their use in high-power and high-frequency applications. Diamond has been applied as a substrate to GaN electronics due to its record-high thermal conductivity. However, optimizing GaN/diamond TBR_eff_ is crucial for efficient heat dissipation in the devices. Previous research focused on modifying the dielectric layer between GaN and diamond, but controlling the TBR has proven to be challenging. In this work, we verified a TBR reduction by a factor of 2 by high-temperature annealing of the SiN_*x*_ dielectric interlayer. Subsequently, we showed that nanopatterning of the diamond to GaN interface can provide major advantages for TBR. By reducing the pitch width, the diamond/GaN effective TBR scaled approximately inversely with interfacial contact area, achieving TBR_eff_ reduction by a factor of 2.6× when the interfacial contact area was increased 4-fold; this was despite the trenches not being fully filled with diamond, and an even greater improvement is possible when fully filling the trenches. Principal stress in GaN, which has a relatively low tensile strength, was simulated to be comparable to or slightly lower in the partially filled diamond/GaN pitches, indicating that thermal stress is not a concern while maintaining the substantial TBR_eff_ reduction achieved with the nanopatterned interface. This work provided a possible solution for the thermal and mechanical management in GaN-on-diamond devices, to be implemented in device applications in the future work.

## Supporting Information Available

Apparatus of ns-TTR; data fitting and sensitivity analysis of SiN_*x*_ TBR_eff_ after high-temperature annealing; data fitting of the diamond/GaN/diamond structure with nanopatterned interface or planar interface; cross-sectional TEM image of the diamond/GaN/diamond structure; and EDS mapping of the diamond/GaN/diamond cross-sectional structure (PDF)

## References

[ref1] Kozak J. P.; Zhang R.; Porter M.; Song Q.; Liu J.; Wang B.; Wang R.; Saito W.; Zhang Y. Stability, Reliability, and Robustness of GaN Power Devices: A Review. IEEE Trans. Power Electron. 2023, 38 (7), 8442–8471. 10.1109/TPEL.2023.3266365.

[ref2] Ballestín-Fuertes J.; Muñoz-Cruzado-Alba J.; Sanz-Osorio J. F.; Laporta-Puyal E. Role of Wide Bandgap Materials in Power Electronics for Smart Grids Applications. Electronics 2021, 10 (6), 677 10.3390/electronics10060677.

[ref3] Pushpakaran B. N.; Subburaj A. S.; Bayne S. B. Commercial GaN-Based Power Electronic Systems: A Review. J. Electron. Mater. 2020, 49 (11), 6247–6262. 10.1007/s11664-020-08397-z.

[ref4] Hoo Teo K.; Zhang Y.; Chowdhury N.; Rakheja S.; Ma R.; Xie Q.; Yagyu E.; Yamanaka K.; Li K.; Palacios T. Emerging GaN technologies for power, RF, digital, and quantum computing applications: Recent advances and prospects. J. Appl. Phys. 2021, 130 (16), 160902 10.1063/5.0061555.

[ref5] Mounika B.; Ajayan J.; Bhattacharya S.; Nirmal D. Recent developments in materials, architectures and processing of AlGaN/GaN HEMTs for future RF and power electronic applications: A critical review. Micro Nanostruct. 2022, 168, 207317 10.1016/j.micrna.2022.207317.

[ref6] Mendes J. C.; Liehr M.; Li C. Diamond/GaN HEMTs: Where from and Where to?. Materials 2022, 15 (2), 415 10.3390/ma15020415. 35057131 PMC8778208

[ref7] Islam N.; Mohamed M. F. P.; Khan M. F. A. J.; Falina S.; Kawarada H.; Syamsul M. Reliability, Applications and Challenges of GaN HEMT Technology for Modern Power Devices: A Review. Crystals 2022, 12 (11), 1581 10.3390/cryst12111581.

[ref8] Zhang Y.; Zubair A.; Liu Z.; Xiao M.; Perozek J.; Ma Y.; Palacios T. GaN FinFETs and trigate devices for power and RF applications: review and perspective. Semicond. Sci. Technol. 2021, 36 (5), 054001 10.1088/1361-6641/abde17.

[ref9] Ma C.-T.; Gu Z.-H. Review of GaN HEMT Applications in Power Converters over 500 W. Electronics 2019, 8 (12), 1401 10.3390/electronics8121401.

[ref10] Husna Hamza K.; Nirmal D. A review of GaN HEMT broadband power amplifiers. Int. J. Electron. Commun. 2020, 116, 153040 10.1016/j.aeue.2019.153040.

[ref11] Pomeroy J. W.; Bernardoni M.; Dumka D.; Fanning D.; Kuball M. Low thermal resistance GaN-on-diamond transistors characterized by three-dimensional Raman thermography mapping. Appl. Phys. Lett. 2014, 104 (8), 083513 10.1063/1.4865583.

[ref12] Zhan T.; Xu M.; Cao Z.; Zheng C.; Kurita H.; Narita F.; Wu Y. J.; Xu Y.; Wang H.; Song M.; Wang W.; Zhou Y.; Liu X.; Shi Y.; Jia Y.; Guan S.; Hanajiri T.; Maekawa T.; Okino A.; Watanabe T. Effects of Thermal Boundary Resistance on Thermal Management of Gallium-Nitride-Based Semiconductor Devices: A Review. Micromachines 2023, 14 (11), 2076 10.3390/mi14112076. 38004933 PMC10673006

[ref13] Chou Y.-C.; Leung D.; Smorchkova I.; Wojtowicz M.; Grundbacher R.; Callejo L.; Kan Q.; Lai R.; Liu P.; Eng D.; et al. Degradation of AlGaN/GaN HEMTs under elevated temperature lifetesting. Microelectron. Reliab. 2004, 44 (7), 1033–1038. 10.1016/j.microrel.2004.03.008.

[ref14] Malakoutian M.; Woo K.; Rich D.; Mandia R.; Zheng X.; Kasperovich A.; Saraswat D.; Soman R.; Jo Y.; Pfeifer T. J. A. E. M.; et al. Lossless Phonon Transition Through GaN-Diamond and Si-Diamond Interfaces. Diamond Interfaces 2025, 11 (1), 2400146 10.1002/aelm.202400146.

[ref15] Soleimanzadeh R.; Naamoun M.; Floriduz A.; Khadar R. A.; van Erp R.; Matioli E. Seed Dibbling Method for the Growth of High-Quality Diamond on GaN. ACS Appl. Mater. Interfaces 2021, 13 (36), 43516–43523. 10.1021/acsami.1c08761. 34464085

[ref16] Feng T.; Zhou H.; Cheng Z.; Larkin L. S.; Neupane M. R. A Critical Review of Thermal Boundary Conductance across Wide and Ultrawide Bandgap Semiconductor Interfaces. ACS Appl. Mater. Interfaces 2023, 15 (25), 29655–29673. 10.1021/acsami.3c02507. 37326498

[ref17] Francis D.; Kuball M. GaN-on-diamond materials and device technology: A review. In Thermal Management of Gallium Nitride Electronics; Elsevier, 2022; pp 295–331.

[ref18] Smith E. J. W.; Piracha A. H.; Field D.; Pomeroy J. W.; Mackenzie G. R.; Abdallah Z.; Massabuau F. C. P.; Hinz A. M.; Wallis D. J.; Oliver R. A.; Kuball M.; May P. W. Mixed-size diamond seeding for low-thermal-barrier growth of CVD diamond onto GaN and AlN. Carbon 2020, 167, 620–626. 10.1016/j.carbon.2020.05.050.

[ref19] Hajek B.; Kohout V.; Flemr V. Note on thermodynamic instability of M 4 C 3-type carbides of gallium group metals. Monatsh. Chem. 1986, 117 (10), 1157–1164. 10.1007/BF00811328.

[ref20] Waller W. M.; Pomeroy J. W.; Field D.; Smith E. J. W.; May P. W.; Kuball M. Thermal boundary resistance of direct van der Waals bonded GaN-on-diamond. Semicond. Sci. Technol. 2020, 35 (9), 095021 10.1088/1361-6641/ab9d35.

[ref21] Zhou Y.; Anaya J.; Pomeroy J.; Sun H.; Gu X.; Xie A.; Beam E.; Becker M.; Grotjohn T. A.; Lee C.; Kuball M. Barrier-Layer Optimization for Enhanced GaN-on-Diamond Device Cooling. ACS Appl. Mater. Interfaces 2017, 9 (39), 34416–34422. 10.1021/acsami.7b08961. 28901127

[ref22] Yates L.; Anderson J.; Gu X.; Lee C.; Bai T.; Mecklenburg M.; Aoki T.; Goorsky M. S.; Kuball M.; Piner E. L.; Graham S. Low Thermal Boundary Resistance Interfaces for GaN-on-Diamond Devices. ACS Appl. Mater. Interfaces 2018, 10 (28), 24302–24309. 10.1021/acsami.8b07014. 29939717

[ref23] Kuzmik J.; Bychikhin S.; Pogany D.; Pichonat E.; Lancry O.; Gaquière C.; Tsiakatouras G.; Deligeorgis G.; Georgakilas A. Thermal characterization of MBE-grown GaN/AlGaN/GaN device on single crystalline diamond. J. Appl. Phys. 2011, 109 (8), 086106 10.1063/1.3581032.

[ref24] Monachon C.; Weber L.; Dames C. Thermal Boundary Conductance: A Materials Science Perspective. Annu. Rev. Mater. Res. 2016, 46 (1), 433–463. 10.1146/annurev-matsci-070115-031719.

[ref25] Giri A.; Hopkins P. E. A Review of Experimental and Computational Advances in Thermal Boundary Conductance and Nanoscale Thermal Transport across Solid Interfaces. Adv. Funct. Mater. 2019, 30 (8), 1903857 10.1002/adfm.201903857.

[ref26] Chen J.; Xu X.; Zhou J.; Li B. Interfacial thermal resistance: Past, present, and future. Rev. Mod. Phys. 2022, 94 (2), 025002 10.1103/revmodphys.94.025002.

[ref27] Wu K.; Chang G.; Ye J.; Zhang G. Significantly Enhanced Interfacial Thermal Conductance across GaN/Diamond Interfaces Utilizing Al_*x*_Ga_*1–x*_N as a Phonon Bridge. ACS Appl. Mater. Interfaces 2024, 16 (43), 58880–58890. 10.1021/acsami.4c13702. 39422442

[ref28] Cho J.; Won Y.; Francis D.; Asheghi M.; Goodson K. E. *Thermal interface resistance measurements for GaN-on-diamond composite substrates* 2014 IEEE Compound Semiconductor Integrated Circuit Symposium (CSICS); IEEE, 2014, pp 1–4.

[ref29] Pomeroy J. W.; Simon R. B.; Sun H.; Francis D.; Faili F.; Twitchen D. J.; Kuball M. Contactless Thermal Boundary Resistance Measurement of GaN-on-Diamond Wafers. IEEE Electron Device Lett. 2014, 35 (10), 1007–1009. 10.1109/LED.2014.2350075.

[ref30] Pomeroy J. W.; Bernardoni M.; Dumka D. C.; Fanning D. M.; Kuball M. Low thermal resistance GaN-on-diamond transistors characterized by three-dimensional Raman thermography mapping. Appl. Phys. Lett. 2014, 104 (8), 083513 10.1063/1.4865583.

[ref31] Sun H.; Simon R. B.; Pomeroy J. W.; Francis D.; Faili F.; Twitchen D. J.; Kuball M. Reducing GaN-on-diamond interfacial thermal resistance for high power transistor applications. Appl. Phys. Lett. 2015, 106 (11), 111906 10.1063/1.4913430.

[ref32] Altman D.; Tyhach M.; McClymonds J.; Kim S.; Graham S.; Cho J.; Goodson K.; Francis D.; Faili F.; Ejeckam F.; Bernstein S. Analysis and characterization of thermal transport in GaN HEMTs on Diamond substrates, Fourteenth Intersociety Conference on Thermal and Thermomechanical Phenomena in Electronic Systems (ITherm), IEEE, 2014; pp 1199–1205.

[ref33] Dumka D. C.; Chou T. M.; Jimenez J. L.; Fanning D. M.; Francis D.; Faili F.; Ejeckam F.; Bernardoni M.; Pomeroy J. W.; Kuball M. Electrical and Thermal Performance of AlGaN/GaN HEMTs on Diamond Substrate for RF Applications, 2013 IEEE Compound Semiconductor Integrated Circuit Symposium (CSICS), IEEE 2013; pp 1–4.

[ref34] Yang S.; Song H.; Peng Y.; Zhao L.; Tong Y.; Kang F.; Xu M.; Sun B.; Wang X. Reduced thermal boundary conductance in GaN-based electronic devices introduced by metal bonding layer. Nano Res. 2021, 14 (10), 3616–3620. 10.1007/s12274-021-3658-7.

[ref35] Cheng Z.; Bai T.; Shi J.; Feng T.; Wang Y.; Mecklenburg M.; Li C.; Hobart K. D.; Feygelson T. I.; Tadjer M. J.; Pate B. B.; Foley B. M.; Yates L.; Pantelides S. T.; Cola B. A.; Goorsky M.; Graham S. Tunable Thermal Energy Transport across Diamond Membranes and Diamond-Si Interfaces by Nanoscale Graphoepitaxy. ACS Appl. Mater. Interfaces 2019, 11 (20), 18517–18527. 10.1021/acsami.9b02234. 31042348

[ref36] Cho J.; Francis D.; Altman D. H.; Asheghi M.; Goodson K. E. Phonon conduction in GaN-diamond composite substrates. J. Appl. Phys. 2017, 121 (5), 055105 10.1063/1.4975468.

[ref37] Liu D.; Francis D.; Faili F.; Middleton C.; Anaya J.; Pomeroy J. W.; Twitchen D. J.; Kuball M. Impact of diamond seeding on the microstructural properties and thermal stability of GaN-on-diamond wafers for high-power electronic devices. Scr. Mater. 2017, 128, 57–60. 10.1016/j.scriptamat.2016.10.006.

[ref38] Koleske D.; Wickenden A.; Henry R.; Culbertson J.; Twigg M. GaN decomposition in H2 and N2 at MOVPE temperatures and pressures. J. Cryst. Growth 2001, 223 (4), 466–483. 10.1016/S0022-0248(01)00617-0.

[ref39] Williams O. A. Nanocrystalline diamond. Diamond Relat. Mater. 2011, 20 (5–6), 621–640. 10.1016/j.diamond.2011.02.015.

[ref40] Cuenca J. A.; Mandal S.; Thomas E. L. H.; Williams O. A. Microwave plasma modelling in clamshell chemical vapour deposition diamond reactors. Diamond Relat. Mater. 2022, 124, 108917 10.1016/j.diamond.2022.108917.

[ref41] Yuan C.; Waller W. M.; Kuball M. Nanosecond transient thermoreflectance method for characterizing anisotropic thermal conductivity. Rev. Sci. Instrum. 2019, 90 (11), 114903 10.1063/1.5099961. 31779394

[ref42] Jiang W.; Xu D.; Xiong B.; Wang Y. Effects of rapid thermal annealing on LPCVD silicon nitride. Ceram. Int. 2016, 42 (1), 1217–1224. 10.1016/j.ceramint.2015.09.053.

[ref43] Francis D.; Vanjari S. C.; Ji X.; Feygelson T.; Spencer J.; Masten H.; Jacobs A.; Lundh J. S.; Tadjer M.; Anderson T. 3D diamond growth for GaN cooling and TBR reduction CS Mantech Conference Proceedings; CS MANTECH, 2024.

[ref44] Lee E.; Zhang T.; Yoo T.; Guo Z.; Luo T. Nanostructures Significantly Enhance Thermal Transport across Solid Interfaces. ACS Appl. Mater. Interfaces 2016, 8 (51), 35505–35512. 10.1021/acsami.6b12947. 27983798

[ref45] Lee E.; Zhang T.; Hu M.; Luo T. Thermal boundary conductance enhancement using experimentally achievable nanostructured interfaces - analytical study combined with molecular dynamics simulation. Phys. Chem. Chem. Phys. 2016, 18 (25), 16794–16801. 10.1039/C6CP01927G. 27275647

[ref46] Zhou X. W.; Jones R. E.; Kimmer C. J.; Duda J. C.; Hopkins P. E. Relationship of thermal boundary conductance to structure from an analytical model plus molecular dynamics simulations. Phys. Rev. B 2013, 87 (9), 094303 10.1103/physrevb.87.094303.

[ref47] Hu M.; Zhang X.; Poulikakos D.; Grigoropoulos C. P. Large “near junction” thermal resistance reduction in electronics by interface nanoengineering. Int. J. Heat Mass Tran. 2011, 54 (25–26), 5183–5191. 10.1016/j.ijheatmasstransfer.2011.08.027.

[ref48] Burkhardt P.; Marvel R. Thermal expansion of sputtered silicon nitride films. J. Electrochem. Soc. 1969, 116 (6), 864 10.1149/1.2412081.

[ref49] Cuenca J. A.; Smith M. D.; Field D. E.; C-P. Massabuau F.; Mandal S.; Pomeroy J.; Wallis D. J.; Oliver R. A.; Thayne I.; Kuball M.; Williams O. A. Thermal stress modelling of diamond on GaN/III-Nitride membranes. Carbon 2021, 174, 647–661. 10.1016/j.carbon.2020.11.067.

[ref50] Brown J. J.; Baca A. I.; Bertness K. A.; Dikin D. A.; Ruoff R. S.; Bright V. M. Tensile measurement of single crystal gallium nitride nanowires on MEMS test stages. Sens. Actuators, A 2011, 166 (2), 177–186. 10.1016/j.sna.2010.04.002.

