# Supplementary material for: Thermal Boundary Resistance Reduction by Interfacial Nanopatterning for GaN-on-Diamond Electronics Applications
Source: ACS Appl Electron Mater. 2025 Mar 27;7(7):2939–46. doi: 10.1021/acsaelm.5c00119 (PMC11984094; doi:10.1021/acsaelm.5c00119)
Supplement: Supplementary file 1 — el5c00119_si_001.pdf [file el5c00119_si_001.pdf]

Supporting Information

Thermal boundary resistance reduction by interfacial nanopatterning for GaN-on-diamond electronics applications

Xiaoyang Ji,[1] Sai Charan Vanjari,[1] Daniel Francis,[1,2] Jerome A. Cuenca,[3] Arpit Nandi,[1] David Cherns,[1] Oliver A. Williams,[3] Felix Ejeckam,[2] James W. Pomeroy,[1] Martin Kuball[1,*]

[1]Centre for Device Thermography and Reliability (CDTR), University of Bristol, Bristol BS8 1TL, United Kingdom;

[2]Akash Systems, San Francisco, CA, United States;

[3]Cardiff School of Physics and Astronomy, Cardiff University, Cardiff, United Kingdom.

[*]Corresponding Author: Martin.Kuball@bristol.ac.uk

1. Apparatus of nanosecond transient thermoreflectance (ns-TTR)

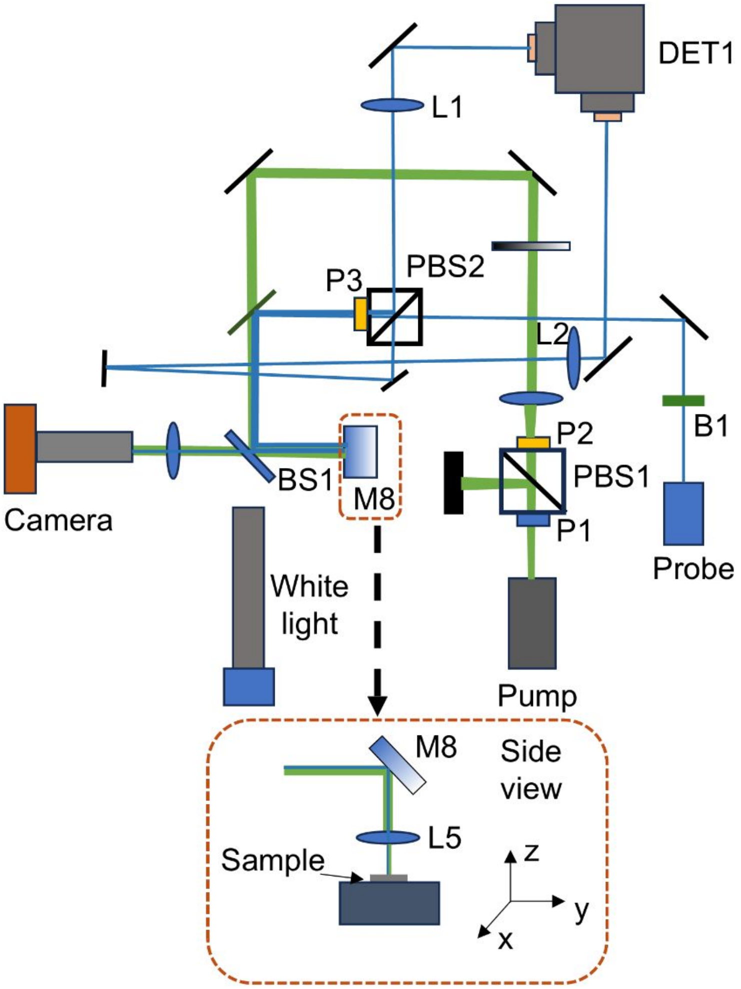

Figure S1. Schematic of nanosecond TTR. B1: beam polarizer; L1, L2: lens, *f*=200 mm; P1: half-wave plate; P2, P3: quarter-wave plate; PBS1, PBS2: polarized beam splitter; BS1: beam splitter; when white light is applied, the beam splitter for laser is replaced by a beam splitter for white light using a flipper mount; M8: mirror to induce a vertical beam direction; L5: lens, *f*=10 mm; DET1: Si photodiode balanced detector.

2. Data fitting and sensitivity analysis of SiN_x TBR_{eff} after high-temperature annealing

The ns-TTR measurements were repeated three times for each sample and sample location, and averaged to improve the signal-to-noise ratio, in all measurements. The three transient curves were processed using a virtual filter, then averaged, and the resulting transient curve was fitted using a numerical solution of the multilayer thermal model. The thermal conductivity of GaN at 1000 °C was determined from a 1000 °C annealed GaN/diamond structure, because the GaN thermal conductivity was obviously affected by high-temperature annealing based on the ns-TTR measurement. The thermal conductivity of GaN is 63.5 W/m-K after annealing at 1000 °C. This suggests that GaN slightly decomposes into Ga and N_2 during the 1000 °C annealing process, despite the densified SiN_x thin film on top of GaN and the use of a full N_2 atmosphere in annealing to prevent potential decomposition.[1, 2] The thermal conductivity of GaN under other annealing conditions remained unaffected, and was fixed at a known value of 130 W/m-K, as reported in previous literature.[3] Additionally, the sensitivity of GaN thermal conductivity is low in the range where the sensitivity of SiN_x thermal conductivity is high. As a result, any uncertainty in the GaN thermal conductivity value does not significantly impact the accuracy of the fitting for SiN_x. These thermal conductivities of GaN were then used as a known property in subsequent fittings of SiN_x/GaN/diamond structures. The effective $1/e^2$ laser beam diameter w_0 on the sample surface was 14.8 μm which was determined from a measurement on a Si standard sample, based on the fitted laser spot size and the Si known thermal conductivity. The determined thermal properties of the materials in the SiN_x/GaN/diamond structures are listed in Table S1. The thermal conductivity of the Cr adhesion layer was determined from TTR measurements of the GaN/diamond substrate (the same substrate as used for the SiN_x/GaN/diamond samples), with this substrate coated with Au/Cr in the same deposition run as the SiN_x/GaN/diamond samples.

Table S1 Typical thermal properties of relevant materials in the multi-layered SiN_x/GaN/diamond structure (for study of TBR_{eff} for impact of SiN_x densification) in the TTR data analysis

Layer #	material	Thermal conductivity (W/m-K)	Specific heat (J/kg-K)	Density (kg/m^3)	Thickness (nm)
1	Au	317	128	19320	165
2	Cr	0.60	450	7140	16
3	SiN_x (deposited)	fitted	800	3190	t*
4	GaN	130/63.5	428	6150	750
5	SiN_x (interlayer in the substrate)	fitted	800	3190	25.5
6	Diamond	1500	509	3500	1000000

* t is the thickness of the annealed SiN_x layer, measured by ellipsometry.

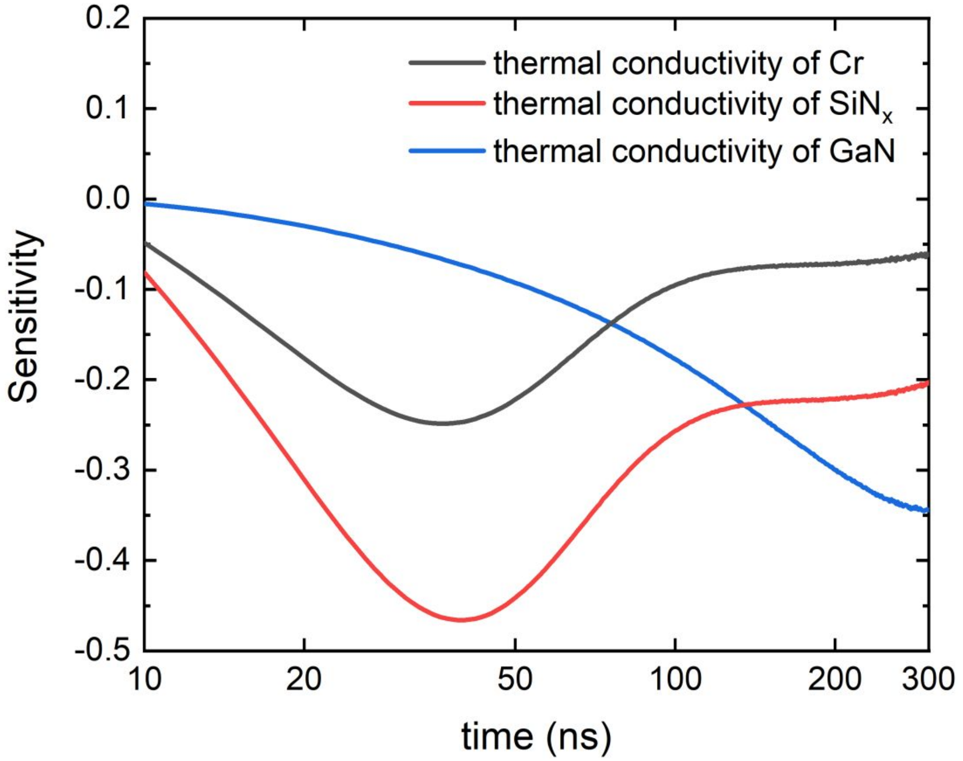

Figure S2. Sensitivity plot of the SiN_x/GaN/diamond structure. In the sensitivity analysis, the thermal conductivity of the annealed SiN_x thin film was set as 1.0 W/m-K and its thickness 25 nm. The thermal conductivity of GaN was set to be 130 W/m-K. The thermal conductivity of the 25-

nm SiN_x layer, located between the GaN and diamond, was taken as 1.05 W/m-K. This sensitivity plot is based on a typical sample annealed at 800 °C. The sensitivities of the corresponding thermal conductivities for samples annealed at 1000 °C are similar.

3.

Table S2 Thermal properties of relevant materials in the diamond/GaN/diamond structure with nanopatterned interface or planar interface

Layer #	material	Thermal conductivity (W/m-K)	Specific heat (J/kg-K)	Density (kg/m^3)	Thickness (nm)
1	Au	317	128	19320	164
2	Cr	0.42/0.50*	450	7140	10
3	Diamond	200	509	3500	850
4	SiN_x (deposited and annealed)	fitted	800	3190	10
5	GaN	63.5	428	6150	750
6	SiN_x (interlayer in the substrate)	1.2	800	3190	25.5
7	Diamond	1500	509	3500	200000

*The Cr thermal conductivity, i.e. folded into the Au/diamond TBR, was taken as 0.42 W/m-K for nanopatterned diamond/GaN/diamond structures and 0.5 W/m-K for the planar interface due to different contact conditions between Cr and the diamond top surface. The values were derived from the data fitting of one or two measurements, and then applied to the other measurements for nanopatterned or planar diamond/GaN/diamond structures. (The Au/diamond TBR is thought to be constant within micro-scale area.)

The effective $1/e^2$ beam diameter w_0 was taken as 30 μm, determined from the measurement of the spot size by camera on the sample surface and the data fitting near 1 μs in the transient curve. The w_0 is larger than the value derived from the Si standard sample, because the surface of diamond/GaN/diamond samples is rougher than the Si surface, and the laser spot size is observed to be larger.

4.

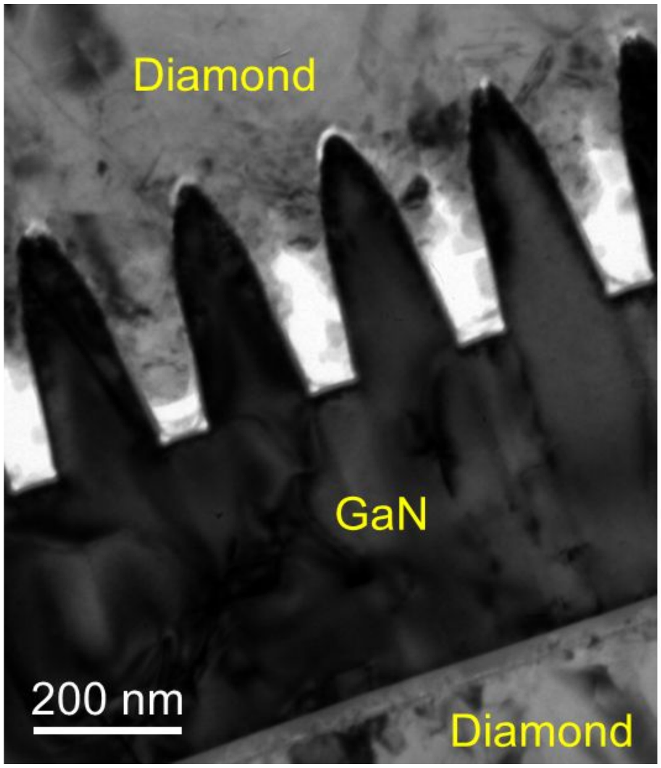

Figure S3. Cross-sectional transmission electron microscopy (TEM) image of the diamond/GaN/diamond structure, with nominal 200-nm pitch.

5. X-ray energy dispersive spectroscopy (EDS) mapping of diamond/GaN/diamond cross-sectional structures

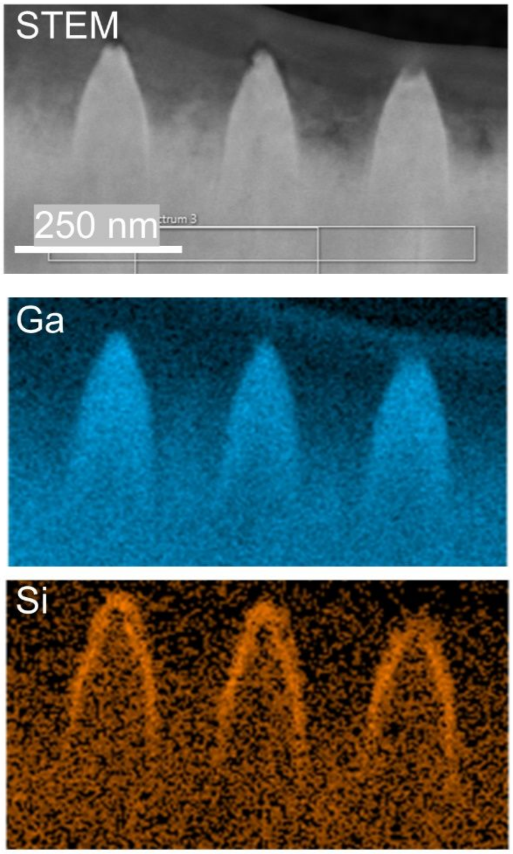

Figure S4. EDS mapping of the diamond/GaN/diamond structure with 200-nm pitch. The cross-sectional lamella was produced by ThermoFisher Scios 2 DualBeam focused ion beam (FIB) milling from the sample with 200-nm pitch. A 30 kV, 30 nA, and 30 kV, 15 nA chunk cutting sequence was used, followed by cleaning at 30 kV 3 nA, 30 kV 50 pA, thinning of the lamellae at 30 kV 0.5 nA, and 30kV 0.3 nA. Finally a low 10 kV polishing was used to obtain the lamellae without any damage and impurity. The EDS mapping was obtained in JEOL TEM with a voltage of 200 kV.

References:

1. Groh, R.; Gerey, G.; Bartha, L.; Pankove, J. I., On the thermal decomposition of GaN in vacuum. *Physica Status Solidi (a)* **1974,** *26* (1), 353-357.

2. Liu, S. S.; Stevenson, D. A., Growth Kinetics and Catalytic Effects in the Vapor Phase Epitaxy of Gallium Nitride. *Journal of The Electrochemical Society* **1978,** *125* (7), 1161.

3. Zhou, Y.; Anaya, J.; Pomeroy, J.; Sun, H.; Gu, X.; Xie, A.; Beam, E.; Becker, M.; Grotjohn, T. A.; Lee, C.; Kuball, M., Barrier-Layer Optimization for Enhanced GaN-on-Diamond Device Cooling. *ACS Applied Materials & Interfaces* **2017,** *9* (39), 34416-34422.
